# Study protocol for the online adaptation and evaluation of the ‘Reboot’ (Recovery-boosting) coaching programme, to prepare critical care nurses for, and aid recovery after, stressful clinical events

**DOI:** 10.1186/s40814-022-01014-2

**Published:** 2022-03-17

**Authors:** K. S. Vogt, A. Grange, J. Johnson, J. Marran, L. Budworth, R. Coleman, R. Simms-Ellis

**Affiliations:** 1 Bradford Institute for Health Research, Bradford Royal Infirmary, Temple Bank House, Duckworth Lane, Bradford, BD9 6RJ UK; 2 Department of Psychology, University of Leeds, Leeds, LS2 9JT UK; 3 School of Public Health and Community Medicine, University of New South Wales, Sydney, 2052 Australia

**Keywords:** Nurses, Critical care, Resilience, Burnout, Healthcare staff, COVID-19, Coaching

## Abstract

**Background:**

Critical care nurses (CCNs) are routinely exposed to highly stressful events, exacerbated during the COVID-19 pandemic. Supporting resilience and wellbeing of CCNs is therefore crucial to prevent burnout. One approach for delivering this support is by preparing critical care nurses for situations they may encounter, drawing on evidence-based techniques to strengthen relevant psychological coping strategies. As such, the current study seeks to tailor a Resilience-boosting psychological coaching programme [Reboot] for CCNs, based on cognitive behavioural therapy (CBT) principles and the Bi-Dimensional Resilience Framework (BDF), and (1) to assess the feasibility of delivering Reboot via online, remote delivery to CCNs, and (2) to provide a preliminary assessment of whether Reboot could increase resilience and confidence in coping with adverse events.

**Methods:**

Eighty CCNs (*n*=80) will be recruited to the 8-week Reboot programme, comprised of two group workshops and two individual coaching calls. The study uses a single-arm before-after feasibility study design and will be evaluated with a mixed-methods approach, using online questionnaires (all participants) and telephone interviews (25% of participants). Primary outcomes will be confidence in coping with adverse events (the *Confidence* scale) and resilience (the *Brief Resilience Scale*) measured at four time points.

**Discussion:**

Results will determine whether it is feasible to deliver and evaluate a remote version of the Reboot coaching programme to CCNs, and will indicate whether participating in the programme is associated with increases in confidence in coping with adverse events, resilience and wellbeing (as indicated by levels of depression).

## Introduction

Over the past 2 years during the COVID-19 pandemic, pressures on healthcare staff have taken on unprecedented levels. The pandemic has required healthcare staff to go above and beyond, with many staff feeling unable to cope with the demands of their healthcare roles and the daily stressors they are faced with [1]. However, even before the emergence of COVID-19, there were reports that the UK National Health Service (NHS) workforce was in crisis, with around a third of doctors and nurses suffering from burnout, compassion fatigue and significant increases in work-related stress amongst its employees [2–6]. Many of the stressors experienced during the COVID-19 pandemic are in direct relation to the virus and the adaptation of healthcare systems, such as high mortality amongst both patients and colleagues, inadequate provision of personal protective equipment (PPE) and medical supplies, redeployment and adherence to continually evolving infection control measures, as well as the increase in extreme physical and emotional stress placed on healthcare workers [1, 7–12]. However, many stressors are also indirectly related to COVID-19: even with better treatment options for the virus and vaccine rollout, cancelled/rescheduled surgeries and late diagnoses (often owing to lack of help-seeking) will now significantly negatively impact staff and resources [13–16]. Thus, increased workloads are likely to persist for an indefinite period. In addition, the ‘knock-on’ effects of COVID-19 on other aspects of healthcare are not yet completely understood, which includes the effects of long Covid on healthcare staff and future patients (e.g. [17–19]).

It is not just during a pandemic that healthcare systems rely on their staff to be resilient and to quickly ‘bounce back’ from stressful experiences or situations, ensuring healthcare systems continue to work effectively. Healthcare is regarded as one of the most stressful professions [20], largely owing to close human contact, the involvement with death and dying people, being exposed to potentially dangerous substances, quick decision-making and the potential impact that this can have on the patient and the risk of making errors (e.g. incorrect medication/dose being prescribed) [21–25]. Statistics from the European Union suggest that medical errors (and arising adverse events) occur in 8–12% (~1 in 10) of patients who are hospitalised [26, 27]. While medical errors can undoubtedly harm the patient, recently, there has been an increasing focus on the impact of these errors on healthcare professionals themselves [28–31]. Involvement in errors has been shown to lead to reductions in clinician wellbeing, disengagement from work and reduced empathy for patients, which in turn may increase the risk of subsequent errors [2, 32–35].

The combination of healthcare professionals’ extremely stressful working environments, with its distinct stressors, and the inevitability of human error make it essential that healthcare professionals are prepared for these situations and that they are able to draw on evidence-based strategies to help process these. As such, Johnson et al. [4] developed a recovery-boosting [*Reboot*] coaching programme, aimed at preparing healthcare professionals for involvement in stressful healthcare events and piloted this on 66 healthcare workers and students(e.g. midwives, specialty doctors, and paramedics). The programme consisted of one 3.5-h workshop and one coaching phone call (60 min). Using the Kirkpatrick model for assessing training interventions, four levels of outcome data were collected (level 1: reaction/opinions, 2: learning, 3: behaviour and 4: results). Outcomes included Confidence in Coping with Adverse Events Questionnaire and the Brief Resilience Scale. At follow-up, participants showed significantly higher levels of psychological resilience and confidence in coping with adverse events, suggesting the intervention was feasible and acceptable to participants and potentially effective for increasing resilience.

Since the onset of the COVID-19 pandemic, the need for psychological support for healthcare professionals has increased, but the feasibility of delivering in-person interventions has reduced. In order to address this gap, the current study will adapt this previously developed resilience coaching programme [4] for remote delivery and evaluate it in critical care nurses (CCNs).

The CCNs are registered nurses, who have the “right knowledge, skills, and competencies to meet the needs of a critically ill patient without direct supervision” [36]. As such, CCNs are an extremely vulnerable group for being routinely exposed to highly stressful events in their daily work, even more so during the COVID-19 pandemic as a clinical area that has been disproportionately affected. Their tasks are varied but centre around management and observation of critically ill patients as well as the provision of psychological support to these patients and their families [37]. The CCNs are adjusting to higher nurse-to-patient ratios (from 1:1 to 1:6+), more rapid patient deterioration and increased provision of end-of-life care. Further tasks include undertaking procedures which are painful for patients, end-of-life issues, dealing with distressed families and providing post-mortem care. This highly stressful nature of CCN work has intensified during the COVID-19 crisis [10, 37, 38]. With around 5–10% of COVID-19 patients needing intensive care, a “dedicated and highly demanding response” [37] has been required of CCNs, causing increased CCN exposure to particularly intense and distressing clinical events [10, 37]. They are standing in for family members, facilitating emotionally distressing remote communication and have absorbed responsibility for training and supervising redeployed, inexperienced/out-of-practice staff [10]. Working in highly infectious conditions, many CCNs have felt conflicted between ethical obligations and primal instincts to protect themselves and their loved ones [39, 40]. Pre-existing staff shortages have compounded these extreme demands [41]; before the pandemic, 43,000 nursing posts stood vacant [42]. High staff stress levels not only damage staff wellbeing, they impact on patient care, leading to poor quality, less safe care [37, 38].

However, CCNs with higher resilience are 18–50% less likely to experience post-traumatic stress disorder (PTSD) through their work than those with lower resilience [43]. Therefore, in both routine work and especially during this pandemic, supporting CCN resilience and wellbeing is crucial. The proposed project tackles CCN stress via a successful resilience-focused psychological coaching programme (*Reboot*). Resilience is the capacity to maintain emotional equilibrium during difficult experiences [44]. Resilience interventions proactively develop the psychological skills to enhance resilience, but resilience training is contentious in healthcare. Some view it as compensating for organisational problems by enhancing staff ‘hardiness’ [45–47].

The negative perception of resilience interventions is compounded by their broad focus on creating *general* coping skillsets. These are usually ‘off-the-shelf’ and do not address the unique stressors faced by healthcare staff. The intervention to be evaluated here (Reboot) addresses many of these criticisms: First, instead of focusing on developing generic coping skills, Reboot targets the stressful clinical events encountered by staff which cannot be addressed by organisational interventions, for which resilience interventions are crucial [5, 44]. These include, for example, managing sudden deaths, communicating bad news to patients, treating distressed or aggressive patients and involvement in patient safety incidents and adverse events [45, 46, 48]. Second, Reboot’s materials are tailored to each disciplinary group, to ensure occupational relevance. Reboot also differs from other psychological support provided during COVID-19 by being *pro-active*, preparing healthcare professionals *before* stressful events occur;* structured*, combining group and individual elements; and *evidence-based*, drawing on both psychological resilience literature [44] and Cognitive behavioural therapy (CBT) techniques [48].

### Rationale and objectives

This study will adapt a previously developed resilience coaching programme [4, 49] for online, remote delivery for CCNs in order to facilitate and enhance access to the programme during the pandemic.

### Primary feasibility objective

The *primary* objective is to assess the feasibility of delivering Reboot via online, remote delivery to CCNs. This will be measured via demand [i.e. how many nurses sign up to the workshops], recruitment [i.e. how many nurses consent to the study] and retention statistics [i.e. how many nurses complete the workshops and coaching calls].

### Secondary objective

The *secondary* objective is to provide a preliminary assessment of whether Reboot could potentially increase both self-reported psychological resilience and confidence in coping with adverse events, via analysis of questionnaires and interviews.

## Methods

### Study design and settings

The study uses a single-arm before-after feasibility study design and will be evaluated with a mixed-methods approach, using online questionnaires (all participants) and telephone interviews (25% of participants). The study duration is 12 months, with 6 months for recruitment and delivery of the coaching programme. The Kirkpatrick model for assessing training interventions [50] will be used, and four levels of outcome data will be collected, as per Johnson et al. [4] (Table 1).

**Table 1:**
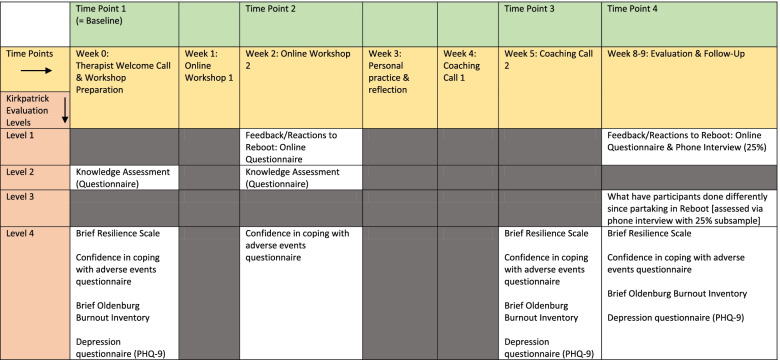
Evaluation of Reboot, using the Kirkpatrick Evaluation Levels

Ethics approval was granted by the University of Leeds Ethics committee (PSYC 302, date: 25-08-2021).

### Participants and inclusion/exclusion criteria

We aim to recruit 80 CCNs, which will allow us to estimate a retention rate of 80% to within a 95% confidence interval ± 8.8% [51, 52] for a potential larger study; that is, we will be able to determine with good confidence whether a large trial could retain a sufficient number of participants (i.e. assessing whether it does lead to large drop-out). In any case, a sample size of 80 is higher than most feasibility projects, which will make our feasibility study more robust and conclusive [53].

Participants must be qualified, registered nurses currently working in critical care settings in the UK.. Participants who are not currently working in critical care are not eligible to participate. However, to ensure participation is confidential and not shared with participants’ employers, participants will not have to demonstrate their eligibility to participate by providing identification or confirmation of employment.

Participants will self-select to partake by responding to advertisements/study flyers circulated by the regional/local critical care network and on social media. One regional critical care network, made up of six Trusts, will be especially supporting recruitment by sustained (re-) advertisement over the recruitment period.

Participants can self-select the timing of when their intervention will start and take place from a list of provided cycles on their sign-up form [“*cycles*” explained in detail below].

### Intervention: adaptation to online, remote delivery

#### Background to the intervention

The current intervention is based on previous work conducted by the team (see Johnson et al. [4]) and draws on cognitive behavioural therapy approaches and psychological resilience theory, namely the Bi-Dimensional resilience Framework (BDF). The BDF suggests that resilience factors are those which reduce the likelihood that exposure to stressors will lead to negative outcomes, such as depression or anxiety [44]. Factors, such as flexible thinking, a positive explanatory style and higher self-esteem, are especially highlighted in this context [33]. As such, the targets of the intervention are threefold: (1) to develop more flexible thinking, including the normalisation of stress and failure, and developing understanding of the interactions between behaviour, mood and cognition; (2) to increase self-esteem when experiencing stress and feelings of failure; and (3) to develop a better explanatory style to increase an individual’s ability to identify, and apply more helpful personal habits in the context of stress and failure (for full targets, see Johnson et al. [4]).

#### Adaptation to online, remote delivery

The format of the initial, in-person face-to-face intervention required adaptation to online, remote delivery. The original study included one in-person, 3.5-h group workshop and one individual coaching phone call (around 60 min) [4]. The following changes were made: the workshop content was changed to two separate small group workshops (120 min each), and the number of coaching calls was doubled from one to two (45–60 min each). In the adapted version for remote delivery, participants are also asked to watch a video prior to their first workshop, explaining the principles of CBT. All workshops and coaching calls will be conducted by an experienced psychologist or CBT therapist. Workshops will be conducted via video conferencing, and coaching calls either via video call or phone call (participants’ preference).

**Fig. 1:**
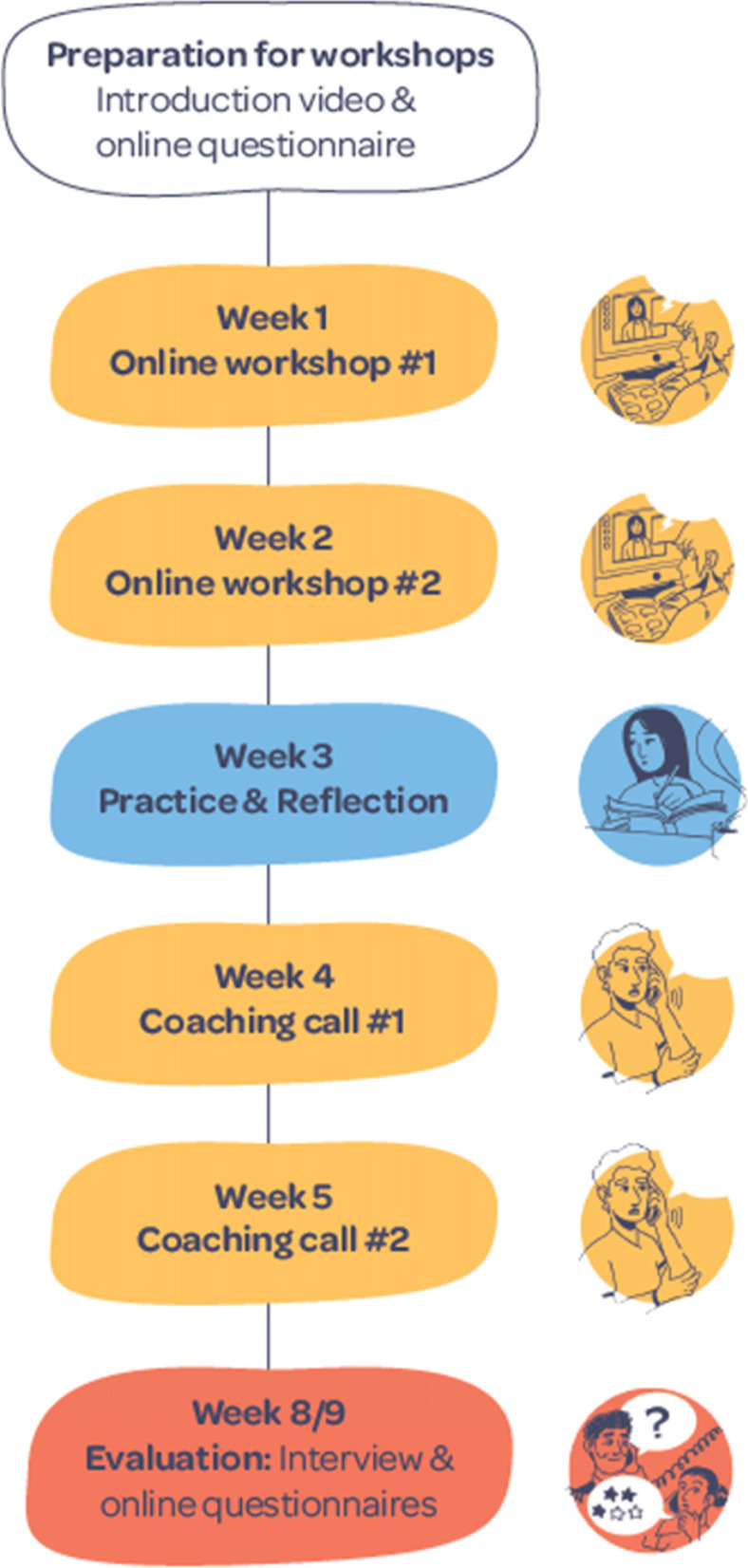
Roadmap for Reboot participants

The study duration will be around 8–9 weeks per participant (from baseline to follow-up); Table 1 outlines the timeline and Fig. 1 is the roadmap provided to participants to illustrate study flow. Workshop dates are organised in cycles; as such, twenty of these cycles will be scheduled. Cycles are numbered 1–20, within which dates/times of workshops are pre-arranged, but dates/times for coaching calls are flexible and arranged directly between the participants and the therapist following workshop 2. Every cycle is limited to 4–6 participants, as previous work indicated that smaller groups perceived more benefits of the intervention (Johnson et al. [4]). Participants will be able to select their preferred cycle.

#### Demographics

A demographics questionnaire was developed for the purpose of this study, capturing (1) participant age, (2) gender, (3) number of years of experience as a registered nurse, (4) number of years of experience as a registered nurse working in critical care, (5) whether they are currently partaking in any other wellbeing initiatives (and details of any) and (6) whether they have, in the past, partaken in any other wellbeing initiatives (and details of any).

#### Outcomes

##### Primary feasibility outcomes

To assess the feasibility of delivering Reboot as an online, remote intervention; demand, recruitment and retention will be assessed.

Demand will be measured by the number of sign-ups to the study, i.e. nurses responding to advertisements for the study and booking a place. Recruitment will be measured by the number of nurses consenting to take part in the study via completion of online questionnaires and attendance of the first workshop. Thirdly, retention will be measured by how many nurses complete the whole coaching programme. Success will be determined using the following criteria:

1. *Demand*: Sign-ups ≥80 participants are deemed successful, as this is our target sample size.
2. *Recruitment*: Consent given and attendance at the first workshop by ≥80 participants.
3. *Retention*: Retention rates of ≥90% for completion of both workshops, ≥70% for completion of the two coaching calls and ≥50% for the final follow-up questionnaire were set, to indicate success. These numbers are based on the retention rates in Johnson et al. [4].

##### Secondary objectives

The *secondary* objective is to provide a preliminary assessment of whether Reboot could potentially significantly increase both self-reported psychological resilience and confidence in coping with adverse events, via analysis of questionnaires and interviews. The outcomes selected are modelled, using the Kirkpatrick model for assessing training interventions, and four levels of outcome data will be collected (level 1: reaction/opinions, 2: learning, 3: behaviour, and 4: results) [50] (Table 1).

Brief Resilience Scale

The Brief Resilience Scale (BRS) [54] contains 6 items measuring perceptions of personal resilience (e.g. “I tend to bounce back quickly after hard times”). The scale is answered on a 5-point Likert scale, from ‘Strongly disagree’ to ‘Strongly agree’, with maximum scores of 24 [range 6–24]; higher scores indicate higher resilience. Previously, the scale has been found to have a test-retest reliability of *α* = 0.69 over 1 month and to converge with responses to longer resilience questionnaires. Participants will complete the BRS at three time points (Table 1).

Confidence

Confidence in coping with adverse events will also be a primary outcome for this study. While not all highly stressful events for CCNs include adverse events, adverse events are highly stressful events when encountered and can have a significant impact on nurse wellbeing. Thus, in keeping with Johnson et al. [4], confidence in coping with adverse events will be measured in this study. Confidence will be measured via the *Confidence in Coping with Adverse Events Questionnaire* (‘Confidence’) which has three items, such as “If I was involved in an adverse event for which I thought I held some responsibility I know the things I would do to help manage my stress levels”. It was developed in the context of the original development of Reboot, as no suitable scale or assessment existed. Items are answered on a 4-point Likert scale [“No, not at all”–“Yes, definitely”]. Scores can range from 3 to 12. The higher the score, the higher the confidence. Participants will complete this questionnaire at four time points (Table 1).

Feedback/reactions

Feedback and reactions to the Reboot workshop will be collected via the feedback questionnaire used in the original study (Johnson et al. [4]). Feedback will be collected from participants immediately after the second workshop. Four quantitative measures are scored on a 5-point Likert scale [1 = ‘Strongly Disagree’ to 5= ‘Strongly Agree’, e.g. “I learned skills in the workshop which will be useful in the future” or “I found the workshop engaging”], and the remaining four require a yes/no response to questions and provide room to expand on answers given [e.g. “Were there any aspects of the workshop you did not find useful?”].

Knowledge

The *Knowledge Assessment* (“Knowledge”) will be used; this assessment was developed in the context of the original development study of the resilience coaching programme, as no suitable scale or assessment existed. This questionnaire measures knowledge communicated within the workshop, such as information about resilience, factors that affect resilience, coping strategies and self-knowledge (personal strengths) [4]. Total possible scores can range from 0 to 6, as one free-text question has a total possible score of 2.

Burnout

An abbreviated version of the Oldenburg Burnout Inventory will be used. The items retained in this version are those with the highest factor loadings across the subscales of the Oldenburg Burnout Inventory (Demerouti et al., 2007; 2008). The six items are in statement format [e.g. “I find my work to be a positive challenge”] and answered along a scale of 1 to 4 [1= ‘Strongly Agree’; 4 = ‘Strongly Disagree’]. The higher the scores, the higher the likelihood of burnout in participants.

Patient Health Questionnaire (PHQ-9)

The PHQ-9 is a validated measure, assessing the presence and severity of depression. Its nine items correspond to the DSM-IV diagnostic criteria for depression, such as feelings of hopelessness or suicidal thoughts. Participants are asked about the frequency with which they experience these, such as “feeling down, depressed or hopeless”. Answers are scored from “Not at all” to “Nearly every day” [corresponding Likert scale 0–3]. Scores can range from 0 to 27 from no depression to severe depression [55, 56]. The PHQ-9 is typically used in primary care to screen for the presence of depression and is currently one of the most commonly used measures to assess the psychological impact of the COVID-19 pandemic on healthcare staff [7]. Previous studies have found good to excellent internal reliability of the PHQ-9 (range *α*: 0.86–.9) [55]. Participants will complete the PHQ-9 at three time points (Table 1).

#### Procedure

Study information will be circulated to the CCNs via Critical Care Networks and social media. Advertisements include flyers, tweets, posts on websites and emails. The advertisements, in the format of flyers and posters, will contain the details (via link or QR code) for the website containing study information. This link will contain both a link to Qualtrics [online survey and data collection tool], where participants can sign up for the study, and the email address of the senior research fellow (SRF) on the project (should participants wish to contact SRF before signing up). Once participants have signed up via the Qualtrics link and provided their details, and preferred dates for the workshops, the SRF will send an email discussing the study and officially booking them onto their first online workshop. A week before the workshop takes place, another Qualtrics link will be sent to the participants. This will include an online consent form and an online baseline survey. Those who wish to participate will complete the consent form and baseline measures [time point 1]. The Qualtrics link will include an online consent form, an online baseline survey and a video link. Those who wish to participate will complete the consent form and baseline measures and watch the video, containing background information about the Reboot intervention. Participants will also receive a text, containing the same information. In addition, the day before the first workshop, the therapist facilitating the workshop and coaching calls will call participants to welcome them to the programme.

The workshops will be delivered to groups of 4–6 participants. At the end of the second workshop, the facilitator will book the participants in for their two coaching calls and ask participants to complete a set of online questionnaires [time point 2], which are the final activity in workshop 2.

The coaching calls will either take place via phone or video call, depending on participant preference. After coaching call 2, participants will be completing another set of questionnaires [time point 3], which they will be sent by text and email after the call has finished. Two to 3 weeks after the second coaching call, all participants will be asked to complete a final set of online questionnaires [time point 4], again, sent via email and text, and a subsample of participants will also be invited to take part in an interview.

Any protocol amendments will be communicated to the funder and relevant journals, should these arise.

#### Statistical analyses

Descriptive statistics (e.g. means, frequencies) will be calculated to provide an overview of the sample, including graphs and tables outlining average outcome values over time.

Feasibility outcomes will be determined numerically, via percentage calculations and count data.

Internal consistency metrics of questionnaires (McDonald’s omega; Cronbach’s alpha) will also be produced for all measures. With the caveat of a small pilot study sample size (i.e. low power) and no control group, exploratory inferential analyses will be conducted. In linear mixed models, a categorical *time* indicator will be used as a predictor of outcomes with participants as the cluster factor, allowing assessment between time point average differences in outcomes (i.e. beta coefficients), adjusted for between-participant variance. It will also be explored whether demographic and work characteristics impact any associations by providing a series of increasingly covariate-adjusted models. The proportion of variance in outcomes explained by fixed model effects will be indexed by ‘marginal’ R2, the proportion of variance explained by the whole model ‘conditional’ R2 (as per our previous work). Intraclass correlation coefficients will be provided to assess the proportion of variance in outcomes explained by between-participant differences. Alpha will be set at 5%, and to boost power, one-tailed tests will be used, assuming increases in outcomes over time (in line with previous findings). R studio will be used to perform all analyses, with the *lme4* package used to fit mixed models.

Missing data will be assessed on a case-by-case basis, owing to the small sample allowing for this. If it can be assumed that missing data is random and by chance, mean substitution may be used; in cases of an increased volume of missing data, the participant will be excluded from the final analysis (however, may still be used for drop-out and subgroup analyses, if the data permits). The amount of missing data will also be used to inform the feasibility of using the outcome measures.

#### Qualitative data collection and analysis

Qualitative interviews will be conducted with a subset of participants (25%, identified via random number generation) around 3 weeks after their second coaching call. In the consent form, all participants consent to being interviewed, but only a small sample will be contacted [to ensure generation of a random sub-sample]. The semi-structured interview schedule previously used by Johnson et al. [4] will be used as a guide for the qualitative interview. The schedule investigates perceptions of the concept of resilience in healthcare and feedback on the coaching programme. Interviews will be conducted by the research team, and not by the therapist who delivered the programme. Interviews will be transcribed verbatim and analysed using Thematic Analysis [57].

#### Access to the data

The anonymised data (both quantitative and qualitative) will be stored at Bradford Teaching Hospitals NHS Foundation Trust servers for 10 years. However, only study team members will have access to the data. Data may be requested from the lead author after data collection and publication of results, after reasonable request. Quantitative data from the questionnaires will be deposited to the White Rose data repository two years after study completion, to allow time for publication, and will then be removed from the servers at Bradford Teaching Hospitals NHS Foundation Trust. Data will be kept on the repository for eight years. Qualitative interview data will not be deposited; as despite the anonymization of data, it is possible that individuals who know participants may be able to identify participants from their transcripts. Thus, these will remain to be stored at Bradford Teaching Hospitals NHS Foundation for the duration of the ten years.

## Discussion

In summary, this study will adapt and evaluate a previously developed resilience training programme intervention [4, 49] to online, remote delivery for CCNs. The study involves the piloting of the Reboot programme with CCNs and assessing feasibility for remote delivery with this population. This will determine whether CCNs can be prepared for highly stressful events in the workplace and whether participating in this coaching programme will increase firstly resilience and confidence in coping with adverse events and secondly wellbeing (as indicated by levels of depression).

### Potential barriers to recruitment and uptake

This is an ambitious project for a duration of 12 months; thus, the study is characterised by time pressures to recruit participants and deliver the study aims and objectives. It is likely that barriers to uptake and recruitment will develop over the course of the recruitment period [September to February], especially considering the recruitment period falling within the notorious “winter pressures” time frame [58–60]. Winter pressures refer to the rising demand on services, both across primary and secondary care associated with the spread of cold and flu viruses [60, 61]. In addition, winter pressures are also typically associated with further decreases in wellbeing, higher prevalence of anxiety and depression, increases in fear of errors and fears of unsafe practices and higher prevalence of staff burnout [60]. Recruitment falling within the winter pressures timeframe may mean that nurses do not want to, or feel able to, engage in work-related material outside of their working hours. Alternatively, nurses may welcome a coaching programme and the opportunity to engage in a tailored, evidence-based programme and the opportunity to engage with a trained mental health professional (psychological therapist), to strengthen their resilience and wellbeing.

### Distress protocol

Owing to the nature of the work of CCNs and high levels of stress and pressure CCNs face, there is the possibility that participants may experience distress. As such, the study has a distress protocol in place. In detail, participants will be made aware they can leave the online workshops and coaching calls with the psychological therapist at any time, withdraw from the study at any time and will be provided with details of their local counselling service (or other most appropriate intervention) if they require additional support. The psychological therapist will receive ongoing clinical supervision to ensure the sensitive delivery of the intervention and address any issues arising.

### Steering group

A steering group has been appointed to the study, and the group will meet three times over the course of the project [beginning, mid-point and towards the end]. The steering group consists of critical care experts, clinical psychologists, nurse consultants in critical care and professors of critical care nursing.

### Dissemination policies

The aim of dissemination for the current study will be to inform whether it is feasible to deliver Reboot on an online, remote platform and whether Reboot can aid recovery from stressful events for critical care nurses. The dissemination strategy will involve presentations at critical care network meetings (both local and national), national and international conferences, two open-access peer-review publications (protocol, study findings) and the development of a Reboot manual, allowing others to deliver the intervention (if successful) to CCNs. Only authors of this protocol, alongside other colleagues associated with the Bradford Institute for Health Research and the University of Leeds, will contribute to the analysis of the data and the dissemination.

## Data Availability

Anonymised behavioural data and statistical analysis may be requested from the lead author after data collection and publication of results.

## Abbreviations

BRS: Brief Resilience Scale
CBT: Cognitive behavioural therapy
CCN: Critical care nurses
NHS: UK National Health Service
PHQ-9: Patient Health Questionnaire
PTSD: Post-traumatic stress disorder
Reboot: *Re*covery *boo*s*t*ing coaching programme
SRF: Senior research fellow
